# The association between permit-to-purchase laws and shootings by police

**DOI:** 10.1186/s40621-023-00439-4

**Published:** 2023-06-29

**Authors:** Cassandra K. Crifasi, Julie Ward, Alex D. McCourt, Daniel Webster, Mitchell L. Doucette

**Affiliations:** Department of Health Policy and Management, Center for Gun Violence Solutions, Johns Hopkins Bloomberg School of Public Health, 624 N. Broadway, Suite 593, Baltimore, MD 21202 USA

**Keywords:** Police violence, Shootings by police, Firearms, Firearms policy, Policy evaluation

## Abstract

**Background:**

Fatal and nonfatal shootings by police are a public health issue that warrants additional research. Prior research has documented associations between fatal shootings by police and gun ownership, legislative strength scores, and lax concealed carry weapons laws. Despite research on other firearm-related outcomes, little is known about the impact of permit-to-purchase (PTP) laws on shootings by police. We generated counts of fatal and nonfatal OIS from the Gun Violence Archive from 2015 to 2020. We conducted cross-sectional regression modeling with a Poisson distribution and robust standard errors. In addition to PTP, we included several state-level policies that may be associated with shootings by police: comprehensive background check only (CBC-only) laws, concealed carry licensing laws, stand your ground laws, violent misdemeanor prohibitions, and extreme risk protection orders (ERPO). We controlled for state-level demographic characteristics and included a population offset to generate incidence rate ratios (IRR).

**Findings:**

PTP laws were associated with a 28% lower rate in shootings by police [IRR = 0.72, 95% confidence interval (CI) 0.64–0.81]. Shall Issue (IRR = 1.34, 95% CI 1.17–1.53) and Permitless (IRR = 1.61, 95% CI 1.35–1.91) concealed carry laws and CBC-only laws (IRR = 1.12, 95% CI 1.01–1.25) were associated with higher rates of shootings by police. Stand your ground, violent misdemeanor prohibitions, and ERPO laws were not associated with shootings by police.

**Conclusions:**

Our study found that PTP laws were associated with significantly lower rates of shootings by police. Removing restrictions on civilian concealed carry was associated with significantly higher rates. State-level firearm policies may be a lever to address shootings by police.

## Introduction

Fatal shootings by police are estimated to occur approximately 1000 times per year. These estimates are based off of databases such as The Counted, Fatal Force, Mapping Police Violence, and the Gun Violence Archive (GVA) developed through media sources, police reports, and other data requests. Currently, GVA is the only data source that includes information on both fatal and nonfatal shootings by police.

Prior research has examined the relationship between proxies for gun ownership, state-level gun laws, and fatal shootings by police. Researchers have found that higher rates of gun ownership were associated with increased rates of fatal shootings by police (Hemenway et al. 2019; Nagin 2020). On average, states that made it easier for people to carry loaded, concealed handguns in public experienced increased rates of fatal and nonfatal shootings by police (Doucette et al. 2022). Additionally, states with higher legislative strength scores had lower rates of fatal shootings by police (Kivisto et al. 2017). Legislative strength scores attempt to measure how strong state guns laws are based on the number of policies a state has in place. However, the overall strength of a state’s gun-related legislation does not provide policymakers or other decision makers with concrete information about which laws they might consider to address issues of gun violence. Therefore, it is necessary to also evaluate the specific policies that are associated, or not, with various forms of violence.

Several studies have examined the role of permit-to-purchase (PTP) laws (sometimes also referred to as purchaser licensing laws) on firearm-related deaths. PTP laws require prospective gun purchasers to first obtain a license from state or local law enforcement prior to buying a gun. PTP systems access both federal and state records to conduct a background check, often facilitated by a fingerprint. These systems also provide law enforcement more time to conduct a background check, and require that sellers, both licensed and private, only sell to those with a valid license. Prior research has found PTP laws to be associated with reductions in firearm homicide (Crifasi et al. 2018; Hasegawa et al. 2019; Webster et al. 2014), firearm suicide (McCourt et al. 2020), and fatal mass shootings (Webster et al. 2020). Research has also found that PTP laws were associated with reductions in diversion of guns for use in crime (Crifasi et al. 2017) (e.g., straw purchasing or other illegal purchasing that contributes to the underground gun market). These laws are also associated with fewer law enforcement officers shot in the line-of-duty (Crifasi et al. 2015). To our knowledge, no research has yet assessed the association of PTP laws and shootings by police.

Given the robust research documenting public safety benefits of PTP laws generally and protecting police officers from being shot in the line-of-duty specifically, we sought to examine whether they were also associated with fatal and nonfatal shootings by police while accounting for other firearm-related policies.

## Methods

Due to the limited availability of high-quality data to assess this outcome, we conducted a cross-sectional analysis to assess the impact of PTP and other firearm laws on fatal and nonfatal shootings by police.

### Data and measures

We compiled counts of fatal and nonfatal shootings by police from the GVA from 2015 to 2020. The GVA has been used previously to study fatal and nonfatal shootings by police (Doucette et al. 2022). We downloaded all incidents classified in the GVA as being an ‘officer-involved incident’ occurring between 2015 and 2020. We reviewed each incident to exclude those where only law enforcement officers were shot as part of an incident or when an officer fired shots, but no one was wounded or killed. We combined fatal and nonfatal shootings by police into one outcome to increase our sample size for the analysis.

In addition to PTP laws, we examined stand your ground (SYG) laws, comprehensive background check laws without a purchaser licensing system (CBC-only), violent misdemeanor prohibitions, extreme risk protection orders (ERPO), and concealed carry weapons laws. Concealed carry laws vary across states but generally are grouped into three categories: May Issue, Shall Issue, and Permitless). May Issue laws are those that have a set of objective criteria for obtaining a license but also require an individual who wants to carry a loaded, concealed handgun in public to have a good and substantial reason or they can be denied. Shall Issue laws also have objective criteria with varying degrees of criminal history or training requirements, but no discretion. Permitless concealed carry allows for anyone not legally prohibited from owning a gun to carry a loaded, concealed handgun in public without an application, background check, or training. Variation in policies was present across states. States were coded as ‘1’ if a policy was in place and ‘0’otherwise. State laws were coded based on prior legal research conducted by our study team (Doucette et al. 2022; Crifasi et al. 2018).

We controlled for several state-level characteristics that, based on the socioecological model and prior research, may confound the relationship between PTP laws and shootings by police: percent of the population living in a metropolitan statistical area (MSA), percent of the population who were black males aged 15–19 years, percent of the population who were white males aged 15–19 years, unemployment rate, per capita alcohol consumption, incarceration rate, law enforcement officers per capita, and rates of violent crime. The incarceration rate and law enforcement officers per capita were lagged 1 year. PTP laws are designed to better identify and screen out individuals prohibited from legally purchasing guns. Therefore, we did not include a proxy for state-level gun ownership as it is likely in the causal pathway between our primary policy variable (PTP) and our outcome (fatal and nonfatal shootings by police).

### Analysis

To estimate the effect of PTP and other firearm laws, we collapsed counts of fatal and nonfatal shootings by police by half-year time periods. The data were indexed by state for a total of 600 state-half-year observations from 2015 to 2020. We assessed for and did not find substantive overdispersion in the data. We ran both negative binomial and Poisson distribution models and found no differences in the point estimates. As a result, we present here the findings from the cross-sectional Poisson distribution model with robust standard errors and a population offset to generate incidence rate ratios (IRR) and 95% confidence intervals (95% CI). We included the policy variables and covariates detailed above in one model to estimate adjusted IRRs. The analysis was conducted using Stata version 17.0. This research was deemed not human subjects by the Johns Hopkins Bloomberg School of Public Health Institutional Review Board.

## Results

PTP laws were associated with a 28% lower rate of shootings by police relative to there being no PTP law (IRR = 0.72, 95% CI 0.64–0.81) (Table 1). Shall Issue (IRR = 1.34, 95% CI 1.17–1.53) and Permitless (IRR = 1.61, 95% CI 1.35–1.91) concealed carry laws were both associated with significantly higher rates of shootings by police. CBC-only laws were also associated with a higher rate of shootings by police, though the 95% CI was close to 1.00 (IRR = 1.12, 95% CI 1.01–1.25). Violent misdemeanor prohibitions, stand your ground laws, and extreme risk protections orders were not associated with shootings by police.

**Table 1:** Cross-sectional analysis examining the associations between firearm laws and fatal and nonfatal shootings by police, 2015–2020

Policy	IRR^a^	*p*-value	95% confidence interval (CI)
Permit-to-purchase	**0.72**	< 0.001	0.64, 0.81
Comprehensive background check only	**1.12**	0.029	1.01, 1.25
Shall issue*	**1.34**	< 0.001	1.17, 1.53
Permitless*	**1.61**	< 0.001	1.35, 1.91
Stand your ground	0.93	0.098	0.86, 1.01
Violent misdemeanor prohibitions	1.02	0.576	0.93, 1.13
Extreme risk protection orders	0.98	0.663	0.88, 1.08

^a^IRRs with a *p*-value < 0.05 are indicated in bold. *‘May Issue’ as reference in three-level categorical variable. The model included the following covariates indexed by state-half-year: unemployment rate, alcohol consumption rate, incarceration rate, rate of law enforcement employment, and percent state living in metropolitan statistical area, black males aged 15–20 years, and white males aged 15–20 years. The model included robust standard errors and had 600 state-half-year indexes

## Discussion

Our analysis found significantly lower rates of shootings by police in states with PTP laws and significantly higher rates in states with fewer restrictions on civilian concealed carry after controlling for factors associated with lethal violence. These findings are consistent with prior research demonstrating protective effects of PTP laws (Crifasi et al. 2018; Hasegawa et al. 2019; Webster et al. 2020). PTP laws intervene at the point of acquiring a handgun. All purchasers are required to first get a license and all sellers, both licensed and private, can only legally sell to someone with a valid license. As a result, these laws provide a more robust system for identifying and screening out prohibited individuals, can deter straw purchasing, or delay impulsive acquisition of a firearm (Crifasi et al. 2017; Webster et al. 2013) all of which can impact the availability of firearms during the commission of a crime or when interacting with law enforcement. Even after controlling for the presence of other firearm laws, our findings are consistent with prior research finding generally harmful effects of lowering standards for concealed carry (Doucette et al. 2022). As states make it easier for people to legally carry loaded, concealed firearms in public, this could increase the likelihood that officers engage with more people who are armed and anticipate that more encounters will involve individuals with firearms (Donohue et al. 2022). Additionally, removing the permitting requirement entirely may increase access to guns via theft from cars and more illegal gun carrying due to reductions in proactive policing on guns (Donohue et al. 2022). More research is needed to understand the interplay between expanded gun access and carrying and shootings by police. For example, survey and/or qualitative work to understand how these policies impact officer perceptions is a necessary piece to understanding any causal mechanisms.

Our analysis found that CBC-only laws were associated with higher rates of shootings by police. CBC-only laws may have been passed in response to higher rates of firearm homicide or other firearm violence, which would lead to greater law enforcement activities and increased interactions with potentially armed individuals. Based on prior research finding evidence of an endogenous relationship between CBC-only laws and firearm homicide at the county level (Crifasi et al. 2018), we hypothesize that may be the case in this analysis as well. However, given the cross-sectional nature of this analysis because of limited data availability, we were not able to directly assess for endogeneity in this analysis. It is important to note this study cannot assess the impact of policy change, but this is a finding that warrants additional research with more robust analyses. Importantly, ERPO laws were not associated with higher rates of shootings by police. In many states, despite several eligible petitioners, law enforcement is often the petitioner and/or the group responsible for serving an ERPO (Pallin et al. 2020; Zeoli et al. 2021; Rowhani-Rahbar et al. 2020). Given the relative newness of these policies, more research is needed. However, this analysis finds that the availability of ERPO laws as a tool to temporarily separate people from their firearms during a time of crisis is not associated with greater risk of shootings by police.

This study is among the first to examine the impacts of PTP and other firearm laws on fatal and nonfatal shootings by police. However, it is not without limitations. There are limitations inherent in the outcome data. We were reliant on a database driven by media reports which may represent an undercounting of the true burden of the issue. However, shootings by police have generated increased levels of community and media attention which may reduce this concern. Our study was restricted to a cross-sectional analysis due to the availability of data and a lack of change in our main variable of interest, PTP, across the study period, which prevented us from assessing policy change. Nor could we assess for any endogeneity that has been seen in prior studies of CBC-only laws. However, we controlled for state-level covariates used in prior research and included other firearm-related policies that could have been associated with our outcome to address selection bias. We did not include a proxy of state-level gun ownership because, for some policies included in the study, a gun ownership proxy could mediate the relationship between gun policies and our outcome. This could bias our findings of policy effects. To our knowledge, there are no national, systematically collected data to measure or estimate police–citizen interactions by state. In an attempt to account for police presence, we included measures for law enforcement officers per capita and the violent crime rate. However, these may not be the best measures of police–citizen interactions that might expose individuals to situations that could lead to a shooting by police. Our study also aggregated fatal and nonfatal shootings by police at the state level. However, there may be unique characteristics of communities at smaller geographic units contributing to differences in the outcome that may be missed in aggregate. Future research should examine smaller geographic units to assess for differential policy effects in narrower social and policy contexts.

Our study found that PTP laws were associated with lower rates of fatal and nonfatal shootings by police. This study adds to the growing body of research suggesting that PTP laws are associated with public safety benefits and a possible policy tool to address multiple forms of violence. PTP laws are a policy strategy decision makers may consider when seeking to implement population-level strategies to address violence including shootings by police.

## Data Availability

The outcome data are not currently available but will be made available in future. Please contact the corresponding author for additional information.

## Abbreviations

CBC: Comprehensive background check only
CI: Confidence interval
ERPO: Extreme risk protection order
GVA: Gun Violence Archive
IRR: Incidence rate ratio
MSA: Metropolitan statistical area
PTP: Permit-to-purchase
SYG: Stand your ground

## References

[CR1] Crifasi CK, Pollack KM, Webster DW (2015). Effects of state-level policy changes on homicide and nonfatal shootings of law enforcement officers. Injury Prev.

[CR2] Crifasi CK, Buggs SAL, Choksy S, Webster DW (2017). The initial impact of Maryland’s firearm safety act of 2013 on the supply of crime handguns in Baltimore. RSF Russell Sage Foundation J Social Sci.

[CR3] Crifasi CK, Merrill-Francis M, McCourt A, Vernick JS, Wintemute GJ, Webster DW (2018). Association between firearm laws and homicide in urban counties. J Urban Health.

[CR4] Donohue JJ, Cai SV, Bondy MV, Cook PJ (2022). More guns, more unintended consequences: the effects of right-to-carry on criminal behavior and policing in US cities. Natl Bureau Econ Res.

[CR5] Doucette ML, Ward JA, McCourt AD, Webster D, Crifasi CK (2022). Officer-involved shootings and concealed carry weapons permitting laws: analysis of gun violence archive data, 2014–2020. J Urban Health.

[CR6] Hasegawa R, Webster D, Small D (2019). Bracketing in the comparative interrupted time-series design to address concerns about history interacting with group: evaluating Missouri's handgun purchaser law. Epidemiology.

[CR7] Hemenway D, Azrael D, Conner A, Miller M (2019). Variation in rates of fatal police shootings across US states: the role of firearm availability. J Urban Health.

[CR8] Kivisto AJ, Ray B, Phalen PL (2017). Firearm legislation and fatal police shootings in the United States. Am J Public Health.

[CR9] McCourt A, Crifasi C, Stuart E (2020). Effects of purchaser licensing and point-of-sale background check laws on firearm homicide and suicide in four states. Am J Public Health.

[CR10] Nagin DS (2020). Firearm availability and fatal police shootings. Ann Am Acad Pol Soc Sci.

[CR11] Pallin R, Schleimer JP, Pear VA, Wintemute GJ (2020). Assessment of extreme risk protection order use in California from 2016 to 2019. JAMA Netw Open.

[CR12] Rowhani-Rahbar A, Bellenger MA, Gibb L (2020). Extreme risk protection orders in Washington: a statewide descriptive study. Ann Intern Med.

[CR13] Webster D, Vernick J, McGinty E, Alcom T, Webster D, Vernick J (2013). Preventing the diversion of guns to criminals through effective firearm sales laws. Reducing gun violence in America: informing policy with evidence and analysis.

[CR14] Webster D, Crifasi CK, Vernick JS (2014). Erratum to: effects of the repeal of Missouri’s handgun purchaser licensing law on homicides. J Urban Health.

[CR15] Webster DW, McCourt AD, Crifasi CK, Booty MD, Stuart EA (2020). Evidence concerning the regulation of firearms design, sale, and carrying on fatal mass shootings in the United States. Criminol Public Policy.

[CR16] Zeoli AM, Paruk J, Branas CC (2021). Use of extreme risk protection orders to reduce gun violence in Oregon. Criminol Public Policy.

